# Measurement properties of the musculoskeletal health questionnaire (MSK-HQ): a between country comparison

**DOI:** 10.1186/s12955-020-01455-4

**Published:** 2020-06-23

**Authors:** David Høyrup Christiansen, Gareth McCray, Trine Nøhr Winding, Johan Hviid Andersen, Kent Jacob Nielsen, Sven Karstens, Jonathan C. Hill

**Affiliations:** 1 Occupational Medicine, Danish Ramazzini Centre, Regional Hospital West Jutland - University Research Clinic, Herning, Denmark; 2 Department of Clinical Medicine, Health, Aarhus University, Aarhus, Denmark; 3 School of Primary, Community and Social Care, Keele University, Staffordshire, UK; 4 Department of Computer Science; Therapeutic Sciences, Trier University of applied Sciences, Trier, Germany

**Keywords:** Patient reported outcomes, Usability, Psychometrics, Responsiveness, Interpretability

## Abstract

**Background:**

The Musculoskeletal Health Questionnaire (MSK-HQ) has been developed to measure musculoskeletal health status across musculoskeletal conditions and settings. However, the MSK-HQ needs to be further evaluated across settings and different languages.

**Objective:**

The objective of the study was to evaluate and compare measurement properties of the MSK-HQ across Danish (DK) and English (UK) cohorts of patients from primary care physiotherapy services with musculoskeletal pain.

**Methods:**

MSK-HQ was translated into Danish according to international guidelines. Measurement invariance was assessed by differential item functioning (DIF) analyses. Test-retest reliability, measurement error, responsiveness and minimal clinically important change (MCIC) were evaluated and compared between DK (*n* = 153) and UK (*n* = 166) cohorts.

**Results:**

The Danish version demonstrated acceptable face and construct validity. Out of the 14 MSK-HQ items, three items showed DIF for language (pain/stiffness at night, understanding condition and confidence in managing symptoms) and three items showed DIF for pain location (walking, washing/dressing and physical activity levels). Intraclass Correlation Coefficients for test-retest were 0.86 (95% CI 0.81 to 0.91) for DK cohort and 0.77 (95% CI 0.49 to 0.90) for the UK cohort. The systematic measurement error was 1.6 and 3.9 points for the DK and UK cohorts respectively, with random measurement error being 8.6 and 9.9 points. Receiver operating characteristic (ROC) curves of the change scores against patients’ own judgment at 12 weeks exceeded 0.70 in both cohorts. Absolute and relative MCIC estimates were 8–10 points and 26% for the DK cohort and 6–8 points and 29% for the UK cohort.

**Conclusions:**

The measurement properties of MSK-HQ were acceptable across countries, but seem more suited for group than individual level evaluation. Researchers and clinicians should be aware that some discrepancy exits and should take the observed measurement error into account when evaluating change in scores over time.

## Background

Pain in muscles and joints is one of the Western World’s chief public health concerns and a leading cause of reduced functional and working capacity [1]. In order to develop effective treatments, and to evaluate their effects, reliable and valid measurement tools are essential. The health-related consequences of musculoskeletal disorders, such as back and knee pain, can rarely be identified or measured using diagnostic imaging or other objective measurement methods [2–5]. Therefore, patient reported outcomes measures (PROMs) are increasingly used to support the assessment, treatment and monitoring of persons with musculoskeletal disorders [6]. PROMs reveal a person’s perceived symptoms, functional ability and quality of life, and are typically administered via questionnaire. A wide range of questionnaires for musculoskeletal disorders have been developed. However, these questionnaires are typically limited to specific parts of the body, e.g. pain in the shoulder and arm [7], neck and back [8, 9], hip and knee [10]. Although, region-specific questionnaires in specific patient population and clinical trials serves its purpose well, they can often be difficult to administrate for health care professionals (e.g. general practitioners and physiotherapists) in primary care, who see patients with diverse musculoskeletal disorders, often presenting with concurrent pain from several locations [11]. Although, more general musculoskeletal questionnaires exist [12, 13], these are relatively comprehensive in length and therefore less suited to evaluate changes over time, because of the burden of administration and patient completion at multiple time points. In addition, some only cover a small number of health-related domains (e.g. pain and function) [13], and thereby may not be sensitive to the multidimensionality of treatment targets inherent in managing musculoskeletal disorders.

As a consequence, Versus Arthritis (https://www.versusarthritis.org) funded collaboration between the universities of Oxford and Keele to develop the Musculoskeletal Health Questionnaire (MSK-HQ) in 2012 [14]. The MSK-HQ aims to capture patient and clinician prioritised key outcomes across a range of musculoskeletal conditions. The instrument consists of 14 items and covers a number of health-related domains, including symptoms, physical functioning, daily activities and work, physical well-being, confidence to manage symptoms, condition understanding and social activities. Each item on the MSK-HQ is answered on a 5-point verbal rating scale (responses coded from ‘not at all’=4 to ‘extremely’ = 0, except for items 12 ‘understanding condition’ and 13 ‘confidence in managing symptoms’, which have the response options in the reverse order). Scores are summed, ranging from 0 to 56, where 56 is the best possible state of musculoskeletal health. The relatively brief format makes the MSK-HQ more suitable to monitor and compare musculoskeletal health status across various conditions and throughout the clinical pathway. The original version of the MSK-HQ has demonstrated high completion rates, good test-retest reliability and strong convergent validity with reference standards in four different MSK cohorts [14]. However, a questionnaire’s usability and measurement properties cannot necessarily be transferred to other countries, languages and healthcare systems [15–17]. Therefore, careful translation, cross-cultural adaptation, testing and validation are necessary in order to take account any cultural and comprehensibility-related differences between the countries. Although, cross-cultural adaptation and within country evaluation often are performed with PROMs, assessment of measurement invariance with respect to language and patients characteristics, as well as direct comparison of measurement properties across countries are less frequent. The objectives of the present study were to 1) translate and cross-cultural adapt the MSK-HQ for use in a Danish-speaking population, and 2) evaluate and compare measurement properties of the MSK-HQ across Danish (DK) and English (UK) cohorts of patients consulting primary care physiotherapy services with musculoskeletal pain.

## Methods

### Translation and cross-cultural adaptation

We translated the MSK-HQ into Danish in accordance with international standards [18] . This was done in close collaboration with the license holder of the MSK-HQ questionnaire, Oxford University Innovation Ltd. (http://innovation.ox.ac.uk) and a representative of the research group, who developed the questionnaire (JCH). A professional English language translator specialising in medical translation and a bilingual physiotherapist, both Danish native speakers, translated the questionnaire from English into Danish. Subsequently, their translations were compared at a consensus meeting in the project group with participation of both translators. A professional native English-speaking translator back-translated the questionnaire from Danish into English, without any prior knowledge of the original version. The back-translation was compared to the original version. Afterwards, the translation report was reviewed by JCH and approved. Finally, we performed cognitive debriefing interviews with 13 patients with musculoskeletal disorders recruited in one physiotherapy clinic in the Central Denmark Region. The results of the cognitive debriefing were presented and reviewed in the project group and the final version of the questionnaire was completed.

## Design and study populations

The study was a prospective comparative study encompassing two consecutively recruited cohorts of patients with musculoskeletal disorders in primary physiotherapy practice in DK and UK.

### DK cohort

#### Participants

Consecutive adult (≥18 years) consulters referred to physiotherapy with a musculoskeletal disorder were invited to participate in six physiotherapy (PT) clinics in the Central Denmark Region. Participants had to be able to understand and speak Danish well enough to complete questionnaires, with no further inclusion criteria applied. In the period January–July 2017 a total of 180 patients agreed to participate, of whom 27 were excluded due to ‘no show’/cancelations (*n* = 9), withdrawal (*n* = 5), other diagnosis than musculoskeletal (*n* = 2), other reasons (*n* = 4) or did not complete baseline questionnaire (*n* = 7), leaving 153 patients for analysis.

#### Data collection

At first contact, the patient was informed about the project. If the patient agreed to participate, an e-mail with a link to an electronic questionnaire was sent to the patient (baseline). The questionnaire included the Danish versions of the MSK-HQ, the generic EQ-5D-5L [19–21] and validated reference standard measures depending on the pain region from which the patient’s main problem originated; Shortened Disabilities of the Arm, Shoulder and Hand (Q-DASH) [22, 23], Neck and Back disability Indexes (ODI and NDI) [24–26], Pain, stiffness and function modules of Knee injury and Hip Disability Osteoarthritis Outcome Scores (KOOS and HOOS) [27–29]. An appointment was made for a first PT consultation (test-retest) 5–7 days later. Immediately before this appointment, the patient once more completed the questionnaire. Follow-up questionnaires were sent after six and 12 weeks by e-mail. In addition, in retest and follow-up questionnaires, patients were asked to rate the overall change in their condition on a 7-point scale (much better, better, slightly better, unchanged, slightly worse, worse, much worse). A total of 134 patients (88%) completed the test-retest questionnaire (median time interval 6 days [Inter quartile range 3–8 days]). Follow-up questionnaires were completed by 118 patients (77%) at 6 weeks and 128 patients (84%) at 12 weeks. For comparison with the UK cohort only the results of the 12-week follow-up are included in the present study.

### UK cohort

#### Participants

The UK cohort was drawn from the primary care physiotherapy sample used as the original validation cohort for the MSK-HQ. The details of materials and methods have previously been described elsewhere [14]. Briefly, the cohort included 210 consecutive consulters in community musculoskeletal physiotherapy clinics in five UK West-Midlands towns. Of those 166 (78%) with test-retest data were available for the present study.

#### Data collection

Participants completed the English paper version of the MSK-HQ and the EQ-5D-5L index before the start of treatment at the first clinic visit (baseline) and again at the second visit, typically 2 weeks later (test–retest). Follow-up questionnaires were completed at 12 weeks by 133 (80%) patients. A transition question on overall change in the condition on a 5-point scale (much better, slightly better, unchanged, slightly worse, much worse) was completed by patients at test-retest and 12-week follow-up.

### Statistical analysis

#### Descriptive statistics

Descriptive statistics were calculated for all variables and compared between the two cohorts. Sum scores were calculated at all time points and raw scores were calculated if no more than 3 items were missing in the respective score; otherwise, the score was left missing. Possible floor and ceiling effects were examined and such effects were considered to be present if more than 15% of the respondents achieved the highest or the lowest sum score, respectively.

#### Construct validity

As no reference standard measures were collected for the UK primary care cohort [14], we only assessed construct convergent validity for the Danish version of the MSK-HQ. This was done by correlation analyses between the MSK-HQ scores and the relevant reference standard measures scores at baseline (Q-DASH, ODI, NDI and WOMAC scores calculated from KOOS and HOOS). Likewise longitudinal convergent validity was assessed by correlation analyses between MSK-HQ and reference standard measures change scores at 12 weeks. Based on findings from the original validation study of the MSK-HQ [14] and previous literature of health outcome research [30–32], we expected correlations between MSK-HQ and relevant reference standard measures to be at moderate to strong (*r* = ≥ 0.5).

#### Cross-cultural validity and measurement invariance

Measurement invariance according to language, categories of age, pain site, duration of pain, and work status, was assessed by Differential item functioning (DIF) analysis of baseline ratings of the two cohorts. DIF is the assessment of the extent to which items function differently between various groups, when the scores among those groups are corrected for. Uniform dichotomous DIF on the raw scores was assessed in this paper via the Mantel-Haenszel (MH) statistic calculated in the R 3.4.1 [33] package difR [34]. Item purification was used and adjustment for multiple comparisons was made via Holm’s method. The assessment of the effect size for the DIF was made on the ETS Delta scale for the dichotomous categories (i.e., country, duration, work status) [35]. Note that MSK-HQ items were dichotomised such that the two lower impact categories (‘not at all’ and ‘slightly/rarely’) opposed the three higher impact categories. Furthermore, for the assessment of pain location, ‘neck’ was collapsed with ‘back’ as there were too few instances of neck pain to calculate the MH statistic robustly.

#### Measurement error and reliability

As test-retest was administered differently in the two cohorts with respect to time interval and initiation of treatment, we restricted the analysis to patients who reported their condition to be ‘stable’ between administrations. Systematic measurement error between MSK-HQ scores at baseline and retest was analysed by Bland-Altman plot and paired t-test for the DK and UK cohorts. Further random errors were estimated by standard error of measurement (SEM) and minimal detectable change (MDC =1.96 × √2 × SEM) was calculated. Cronbach’s alpha was calculated to assess internal consistency. The intra class correlation coefficient (ICC 2.1) was used to assess test-retest reliability, and for single items Cohen’s Kappa with quadratic weights was used. Confidence intervals (95% CI) for Kappa values were obtained using non-parametric bootstrap methods (1000 replications).

#### Sensitivity to change, responsiveness and interpretability

To evaluate sensitivity to change MSK-HQ change scores from baseline to 12 weeks and effect-size statistics (i.e., mean change/ standard deviation at baseline) were calculated and compared between the two cohorts. For responsiveness the MSK-HQ’s ability to discriminate between unchanged patients was calculated and compared between cohorts by receiver-operating-characteristic (ROC) curve analyses with large improvement (much better, better versus a little better, unchanged, little worse) and small improvement (much better, better, little better versus unchanged), using the transition question as external anchor. Responsiveness to worsening was not analysed, as only few patients rated their condition to be ‘worse’ or ‘much worse’. Minimal clinically important change (MCIC) values were estimated by the Pythagoras’ Theorem (a^2 + b^2 = c^2) to choose the change score closest to the upper left-hand corner, which best discriminated between improved and unchanged patients [36]. As MCIC values can be affected by baseline scores, analysis was repeated with relative change scores (i.e., change scores expressed as percentages of the baseline scores) [37]. Finally, as a key vison for the development of the MSK-HQ was to produce a single musculoskeletal health measure superior to generic health tools, we compared effect size estimates and areas under the ROC curve for the MSK-HQ and the EQ-5D change scores [38]. The statistical package STATA version 15 was used.

## Results

### Translation and cross- cultural adaptation

In the translation process a few items had to be slightly rephrased and the response category ‘severely’ was changed to ‘a lot’ to fit the Danish language. A few other issues were raised during translation. The two different constructs; pain and stiffness used in item 1 and item 2 led to some discussion in the project group, as well as the different constructs in item 12 ‘understanding condition’ and item 13 ‘confidence in managing’. However, cognitive debriefing interviews with pilot patients (five males and eight females, 32–59 years of age) with various musculoskeletal disorders revealed no difficulties with respect to the above-mentioned concerns. In general, patients found the Danish version of the MSK-HQ easy to complete (2–6 min) and items were well understood, thus no further changes were needed. Patients did not seem to distinguish between pain and stiffness, and most patients found the items regarding understanding their condition (item 12) and confidence in managing (item 13) to be important. The Danish version of the MSK-HQ is available on licence request: https://process.innovation.ox.ac.uk/clinical.

### Descriptive statistics

The patient characteristics of both cohorts are presented in Table 1. The two cohorts were fairly similar with respect to distribution of pain sites. The patients in the DK cohort were slightly younger, included more women, and had a higher percentage of patients who were working. No floor or ceiling effects were observed at any time point. In the Danish cohort missing data was replaced by mean item scores for two patients who left two items unanswered and two patients who left three items unanswered at baseline, and two patients who left one item at retest and follow-up. For the UK cohort mean item score was calculated for two patients leaving one item unanswered.

**Table 1:** Patient characteristics

Variables	DK cohort (***n*** = 153)		UK cohort (***n*** = 166)	
**Pain site**				
Back	45	(29.4)	47	(28.3)
Neck	23	(15.0)	7	(4.2)
Upper extremity	38	(24.8)	35	(21.1)
Lower extremity	47	(30.7)	57	(34.3)
Other/unknown	–	–	20	(12.0)
**Gender**				
Woman	97	(63.4)	92	(55.4)
Men	56	(36.6)	74	(45.6)
**Age**				
Years mean, (SD)	50.4	(13.8)	53.7	(15.7)
**Work status**				
Working	96	(62.8)	94	(56.6)
Not working/Subsided job	6	(3.9)	7	(4.2)
Not working/unemployed	14	(9.2)	5	(3.0)
Retired	26	(17.0)	52	(31.1)
On sick leave	11	(7.2)	7	(4.2)
Unknown	–	–	1	(0.1)
**Duration of symptoms**				
≤ 3 months	62	(40.5)	77	(47.2)
> 3 months	91	(59.4)	66	(39.8)
Unknown	–	–	23	(13.9)
**Baseline score**				
MSK-HQ (0 to 56) mean, (SD)	32.3	(9.2)	30.5	(9.6)
EQ-5D-5L index (−0.6 to 1.0) mean, (SD)	0.69	(0.16)	0.55	(0.25)

Values are numbers (percentages) unless stated otherwise

*Abbreviations*: *SD* standard deviation

### Construct validity

The correlation coefficients (Spearman Rho) between the Danish MSK-HQ and relevant reference standard measures were 0.63 (CI, 0.38 to 0.79) for the Q-DASH, 0.71 (CI, 0.53 to 0.88) for the ODI, 0.60 (CI, 0.25 to 0.81) for the NDI, and for the WOMAC 0.84 (CI, 0.73 to 0.91). Corresponding values for change scores were 0.65 (CI, 0.38 to 0.82) for the Q-DASH, 0.74 (CI, 0.54 to 0.86) for the ODI, 0.52 (CI, 0.05 to 0.80) for the NDI, and for the WOMAC 0.70 (CI, 0.50 to 0.83).

### Cross cultural validity and measurement invariance

With regards to country differences, three items showed statistically significant levels of DIF, all with large effect sizes^1^ according to the ETS Delta scale [35], these were items 2 (pain/stiffness at night; lower than expected in UK – MH = 8.54, *p* = 0.04), 12 (understanding condition; lower than expected in DK - MH =11.37, *p* = 0.01), and 13 (confidence in managing; lower than expected in DK - MH = 15.21, *p* = 0.001). With regards to pain location, three items showed statistically significant levels of DIF, items 3, 4 and 5. Item 3 (walking; hardest for lower extremities, moderate for back/neck and easiest for upper extremities - MH = 38.71, adj.*p* < 0.001), item 4 (washing/dressing; harder for upper extremities, easier for lower extremities and back/neck - MH = 11.34, adj. p = 0.04), item 5 (physical activity levels; easier for upper extremities, harder for lower extremities and back/neck - MH = 14.57, adj.p = 0.01). With regards to age group, pain duration and working status, no items exhibited statistically significant DIF.

### Measurement error and reliability

Of 134 patients in the DK cohort and 165 patients in the UK cohort with retest MSK-HQ data available, 98 and 82 patients reported their condition to be ‘stable’ between baseline and retest, respectively and were thus used to calculate the reliability statistics presented in Table 2. When compared to the UK cohort, smaller systematic and random errors were observed in the Danish cohort and ICC values were slightly higher. Internal consistency was high for both cohorts. The median (range) weighted kappa with squared weights across the 14 items for the DK cohort was 0.69 (0.54 to 0.84) as compared to 0.67 (0.31 to 0.80) in the UK cohort. Details of the test-retest reliability of single items of the MSK-HQ are available in Appendix A.

**Table 2:** Measurement error and reliability of MSK-HQ scores for the DK cohort and the UK cohort

	DK-cohort (***n*** = 98)		UK- cohort (***n*** = 82)	
**Measurement error**				
Mean Difference (95% CI)	1.6	(0.7 to 2.5)	3.9	(2.7 to 5.1)
SEM (95% CI)	3.1	(2.7 to 3.6)	3.6	(3.1 to 4.1)
MDC (95% CI)	8.6	(7.6 to 10.1)	9.9	(8.7 to 11.3)
**Reliability**				
ICC (95% CI)	0.86	(0.81 to 0.91)	0.80	(0.50 to 0.90)
Cronbach’s α (Baseline)	0.88	(0.85 to 0.91)	0.89	(0.86 to 0.89)

*Abbreviations*: *CI* Confidence interval, *SEM* Standard error of the measurement, *MDC* Minimal Detectable Change;

### Sensitivity to change, responsiveness and interpretability

**Fig. 1:**
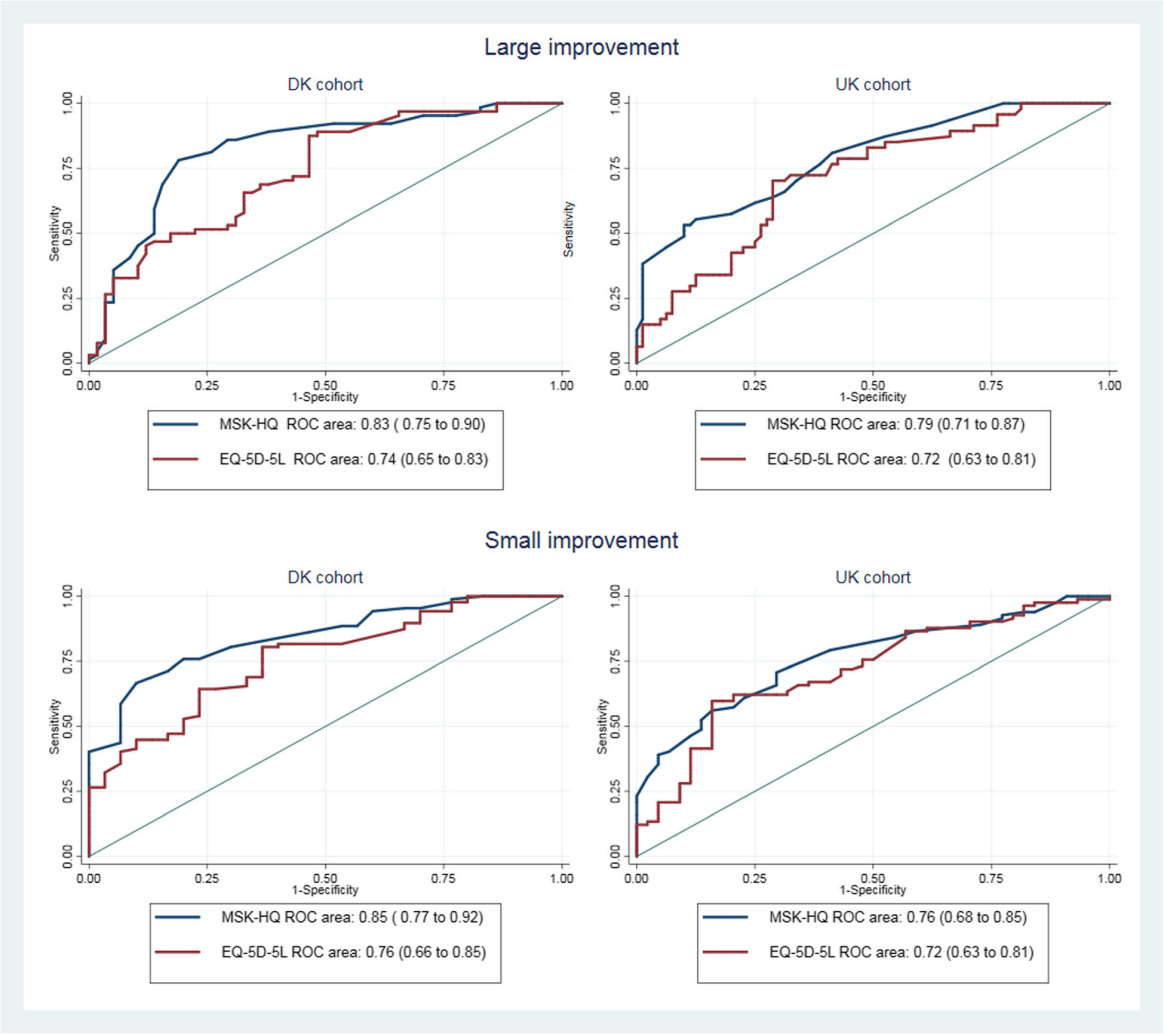
Receiver-operating-characteristic (ROC) curves representing absolute change scores of the MSK-HQ and EQ-5D-5L. Values are ROC area estimates (95% confidence intervals)

At 12 weeks for the DK cohort mean change scores were 9.1 (95% CI, 7.7 to 10.6); ES 1.1 (CI 0.9 to 1.2) for the MSK-HQ and mean EQ-5D-5L index scores were 0.08 (CI, 0.06 to 0.10); ES 0.7 (CI, 0.5 to 0.8). The corresponding values for the UK cohort were mean score 8.9 (CI, 7.4 to 10.5); ES 0.9 (CI, 0.8 to 1.1) for the MSK-HQ and for the EQ-5D-5L index mean score 0.11 (CI, 0.07 to 0.14); ES 0.5 (CI 0.3 to 0.6). The correlations (Spearman Rho) between the transition question of change in main complaint and MSK-HQ change scores were − 0.66 (CI, − 0.75 to − 0.55) and − 0.54 (CI, − 0.65 to − 0.40) for the DK and UK cohorts, respectively. Table 3 presents the ROC analyses and MCIC estimates according to the two cut points of patient perceived important change in main complaint. Although, ROC AUC differed between the DK and UK cohorts, these differences did not reach statistical significance. The MCIC estimate for absolute change at 12 weeks was larger for the DK cohort, whereas for the percentages of change the opposite was true. The percentages reaching the MCIC threshold for ‘large improvement’ were 48% in the DK cohort and 47% in the UK-cohort, whereas the corresponding values for ‘small improvements’ were 57 and 56%, respectively. Figure 1 illustrates the ROC curves representing change scores for the MSK-HQ as compared to EQ-5D-5L index at 12 weeks. For both cohorts the MSK-HQ produced higher ROC AUCs than the EQ-5D-5L. For the DK cohort the difference between MSK-HQ and EQ-5D-5L ROC areas index reached borderline statistical significance for both large improvement (*p* = 0.05) and small improvement (*p* = 0.06).

**Table 3:** ROC and MCIC estimates according to patient perceived important change in main complaint

Cut points	n (%)	ROC_AUC_ (95% CI)	MCIC_ROC_	Sensitivity/ specificity %	MCIC_percent_
**Large improvement**					
DK cohort	64 (52.5)	0.83 (0.75 to 0.90)	10	78/81	26
UK cohort	49 (37.1)	0.79 (0.71 to 0.87)	8	78/69	29
**Small improvement**					
DK cohort	87 (74.3)	0.85 (0.77 to 0.92)	8	76/80	26
UK cohort	85 (64.9)	0.76 (0.68 to 0.85)	6	71/71	29

a) Large improvement (much better, better versus a little better, unchanged, little worse)

b) Small improvements (much better, better, little better versus unchanged)

MCIC_ROC_: Estimated as the optimal cut-off point of the ROC curve using absolute change scores

MCIC_percent_: Estimated as the optimal cut-off point of the ROC curve using relative change scores

*Abbreviations*: *ROC* receiver operating characteristic, *AUC* area under the curve, *CI* confidence interval

## Discussion

This is the first study to evaluate and compare measurement properties of the new MSK-HQ tool across different international cohorts of musculoskeletal patients consulting primary care physiotherapy services. The MSK-HQ was translated and cross-cultural adapted to Danish speaking population. The Danish version of the MSK-HQ demonstrated good face validity and construct validity was confirmed by moderate to strong correlations with relevant reference standard measures. In the DIF analysis three items displayed measurement non-invariance for language (pain/stiffness at night, understanding condition, confidence in managing) and three for pain location (walking, washing/dressing, physical activity levels), whereas no differences were observed for age, pain duration and working status. For both cohorts the MSK-HQ demonstrated good internal consistency, acceptable levels of reliability and responsiveness with reliability coefficients and ROC estimates exceeding 0.70 [15].

The study benefitted from prospectively collected data in two comparable physiotherapy cohorts with high completion rates on questionnaires. The study had some limitations. No formal sample size calculation for the present study was performed, but as both cohorts exceeded the COSMIN recommendations of at least 100 individuals for quantitative PROM studies [15], we believe the sample sizes of the two cohorts to be adequate. It should also be noted that data of the two cohorts was collected and administrated differently. Thus, only limited population descriptors across the two cohorts were available, with data being less complete in the comparative UK cohort for the variables pain site and duration of symptoms. The questionnaires in the UK cohort were paper based whereas questionnaires in the DK cohort were completed online. As studies comparing paper Vs, online administered patient-reported outcome measures indicate these two assessment methods are highly comparable [39], it is unlikely to have affected our results.

The high completion rate, acceptable face and construct validity observed confirmed earlier promising findings from the initial validation study [14]. In terms of reliability and measurements error the DK version seems to perform slightly better than the UK version in our study. We also observed a significantly larger systematic measurement error of test-retest scores of MSK-HQ for the UK cohort. These findings may, in part, be explained by differences in study designs. The DK cohort patients completed test-retest questionnaires with a 6 days interval before any treatment was initiated, whereas patients in the UK cohort completed it within a 2 weeks interval. Although, these differences were levelled out by restricting test-retest analysis to ‘stable’ patients only, it is possible that the longer time interval could have resulted in more improvement among ‘stable’ patients in the UK-cohort. It could be argued that restricting analysis to ‘stable’ patients would inevitably produce lower MDC estimates, than if ‘unstable’ patients were included. However in our case, if all available patients had been included it would only have increased the MDC estimate by approximately one point (results not shown), and therefore not have changed the overall conclusion of the study. Another limitation of the present study was that classification of improved or unchanged patients were based on a single transition scale question as external anchor (i.e. improvement in overall condition), which might measure varying aspects of the outcome [32]. The use of several anchors covering different aspects of the outcome (e.g. pain, function and patient satisfaction) might have produced other results. Furthermore, note that alternative methods to derive MCIC estimates do exist [40, 41] however we chose to use the ROC curve method to preserve comparability with other studies in the field and communicability with readers unfamiliar with alternative methods.

Three MSK-HQ items exhibited DIF across countries. Item 2 ‘pain/stiffness’ at night was scored lower (i.e. more pain/stiffness) in UK patients although no differences were found for the similar item 1 ‘pain/stiffness’ during the day. DK patients had lower item scores with regards to understanding condition and confidence in managing (item 12 and 13). These differences may either imply translational problems, cultural differences or true differences between cohorts. This should be further investigated through qualitative inquiries to explore how patients understand and interpret these questions across countries. Although, these two items were deemed important and relevant by patients, they have previously shown to correlate poorly with the total MSK-HQ score [14]. Hence, it could be discussed whether to include these items in the overall sum score or to use these questions separately to facilitate patient communication and shared clinical decision making. The findings that no MSK-HQ items differed for age, pain duration and working status adds to the pervious results from the validation study on the original version [14]. With regards to pain location, the lower scores found in item 3 ‘walking’ for extremities and back/neck patients, when compared to upper extremity patients was not an unexpected finding, and seems in line with the opposite pattern for item 4 ‘washing/dressing’. It could be speculated that the higher scores found for upper extremities patients for item 5 ‘physical activity levels’, may be due to examples given (going for a walk or jogging), as these do not included physical activities related to upper extremities. Adding riding your bike or playing tennis/golf could be a potential solution to this discrepancy between pain sites.

The distinction between *formative* (i.e., the items cause the construct) and *reflective* (i.e., the construct causes that which is measured by the items) tools is not always easy to make and tools can exhibit aspects of both conceptual frameworks [42]. Note that previously, the MSK-HQ had been considered to be a *formative* tool however on further reflection we feel that tool is much better characterised as being *reflective*. The measurement property estimates observed for the MSK-HQ in the present study did not differ from most existing region-specific PROMs [7, 9, 10, 43] and general musculoskeletal PROMs [44]. For PROMs, reliability coefficients ≥0.7 are considered adequate for group comparisons, whereas ≥0.9 are needed to monitor individual patients [45]. Similar to most musculoskeletal PROMs, the MSK-HQ only exceed the first mentioned threshold, and therefore, at present, may be most suitable for group evaluation. MDC values for musculoskeletal PROMs are commonly reported to range from 10 to 20% of the scale, which is in line with our findings for the MSK-HQ. To ensure that a change score on an individual patient level is clinically relevant, the MCIC should be greater than, or at least equal to, the random measurement error (MDC) of an instrument. This was only true in the Danish cohort of the present study and for the cut-off-level of large improvements, making the interpretability of small changes of individual scores of the MSK-HQ more challenging. As MCIC estimates based on relative scores were unaffected by the cut-off level of the transition question and relative scores have shown to be less sensitive to baseline scores [37]; the MCIC_percent_ seems the preferable choice. However, both absolute and relative MCIC values varied between the two cohorts. These differences may partly be rooted in the use of different scaling of transition question of main complaint (DK cohort 7 versus 5 response categories), where a stronger correlation between the transition question and MSK-HQ change scores at 12 weeks was observed. On the other hand the proportion exceeding MCIC thresholds between the DK cohort and UK cohort did not differ substantially; large improvements (48% versus 47%) and small improvements (57% versus 55%). The MSK-HQ seems to discriminate well between unchanged and improved patients across the two cohorts. A key vision of the MSK-HQ was to produce a single broad health-status measure more sensitive to change than generic health tools. In both cohorts effect sizes of the MSK-HQ were considerably larger than those of the EQ-5D-5L, which indicates superiority of the MSK-HQ. Whereas for the ability of MSK-HQ to discriminate between improved and unchanged (i.e. responsiveness) a patient at 12 weeks, superiority was only observed with respect to the Danish cohort.

## Conclusions

In this study we performed a cross-country comparison of the MSK-HQ questionnaire among musculoskeletal patients consulting primary care physiotherapy services. Although, some discrepancy for language and pain site location was found for single items, the MSK-HQ generally produced comparable results across the two cohorts. The Danish version of the MSK-HQ appears to outperform the original English version and has shown to be a reliable, valid, sensitive and responsive instrument to capture and monitor musculoskeletal health status.

## Data Availability

DK cohort data cannot be made publicly available according to Danish regulations. Data are however available from the authors upon reasonable request and permission of the Danish Data Protection Agency. For the UK cohort additional data can be accessed on request via the Keele data repository at: http://www.keele.ac.uk/pchs/publications/datasharingresources/

## Appendix

**Table 4:** Test-retest reliability of single items of the MSK-HQ for the DK and UK cohort

Item	Question	DK cohort (***n*** = 98)	UK cohort (***n*** = 82)
1	Pain/stiffness during the day	0.73 (0.63 to 0.81)	0.55 (0.40 to 0.67)
2	Pain/stiffness at night	0.58 (0.48 to 0.69)	0.68 (0.55 to 0.78)
3	Walking	0.84 (0.76 to 0.90)	0.74 (0.62 to 0.84)
4	Washing/Dressing	0.64 (0.44 to 0.80)	0.66 (0.51 to 0.78)
5	Physical activity levels	0.76 (0.66 to 0.84)	0.69 (0.56 to 0.79)
6	Work/daily routine	0.82 (0.74 to 0.88)	0.61 (0.48 to 0.71)
7	Social activities and hobbies	0.56 (0.41 to 0.68)	0.59 (0.43 to 0.73)
8	Needing help	0.78 (0.66 to 0.87)	0.78 (0.69 to 0.85)
9	Sleep	0.75 (0.62 to 0.84)	0.80 (0.71 to 0.87)
10	Fatigue or low energy	0.77 (0.64 to 0.85)	0.75 (0.63 to 0.83)
11	Emotional well-being	0.57 (0.43 to 0.71)	0.78 (0.69 to 0.85)
12	Understanding condition	0.54 (0.38 to 0.69)	0.31 (0.13 to 0.48)
13	Confidence in managing	0.58 (0.45 to 0.70)	0.49 (0.25 to 0.69)
14	Overall impact	0.62 (0.47 to 0.73)	0.58 (0.43 to 0.69)

Values are weighted kappa with squared weights (95% Confidence intervals)

## Abbreviations

MSK-HQ: The Musculoskeletal Health Questionnaire
PROM: patient reported outcome
DIF: Differential item functioning
ICC: Intraclass Correlation Coefficients
SEM: standard error of measurement
MDC: minimal detectable change
MCIC: Minimal clinically important change
CI: Confidence intervals
SD: standard deviation

## Footnotes

1 Note: the ETS Delta Scale has three categorisations for DIF; (A) Negligible: non-significant or effect size lower than 1, (B) Intermediate: neither A nor C true, (C) Large: Effect size > 1.5 AND significantly different from an effect size of 1. See the reference for the calculation of the effect size.
